# Hypoxia in relationship to tumor volume using hypoxia PET-imaging in head & neck cancer – A scoping review

**DOI:** 10.1016/j.ctro.2022.06.004

**Published:** 2022-06-15

**Authors:** Sofia Hildingsson, Maria Gebre-Medhin, Sebastian Zschaeck, Gabriel Adrian

**Affiliations:** a Division of Oncology and Pathology, Clinical Sciences, Lund University, Lund, Sweden; b Department of Hematology, Oncology and Radiation Physics, Skåne University Hospital, Lund University, Lund, Sweden; c Department of Radiation Oncology, Charité Universitätsmedizin Berlin, Corporate Member of Freie Universität Berlin, Humboldt-Universität zu Berlin, and Berlin Institute of Health, Berlin, Germany

**Keywords:** Head and neck cancer, Hypoxia, PET-imaging, Tumor volume, Individualized radiation therapy

## Abstract

**Background:**

Hypoxia and large tumor volumes are negative prognostic factors for patients with head and neck squamous cell carcinoma (HNSCC) treated with radiation therapy (RT). PET-scanning with specific hypoxia-tracers (hypoxia-PET) can be used to non-invasively assess hypoxic tumor volume. Primary tumor volume is readily available for patients undergoing RT. However, the relationship between hypoxic volume and primary tumor volume is yet an open question. The current study investigates the hypotheses that larger tumors contain both a larger hypoxic volume and a higher hypoxic fraction.

**Methods:**

PubMed and Embase were systematically searched to identify articles fulfilling the predefined criteria. Individual tumor data (primary tumor volume and hypoxic volume/fraction) was extracted. Relationship between hypoxic volume and primary tumor volume was investigated by linear regression. The correlation between hypoxic fraction and log_2_(primary tumor volume) was determined for each cohort and in a pooled analysis individual regression slopes and coefficients of determination (R^2^) were weighted according to cohort size.

**Results:**

21 relevant articles were identified and individual data from 367 patients was extracted, out of which 323 patients from 17 studies had quantifiable volumes of interest. A correlation between primary tumor volume and PET-determined hypoxic volume was found (*P <.001, R^2^ = 0.46).* Larger tumors had a significantly higher fraction of hypoxia compared with smaller tumors (*P<*.01). The weighted analysis of all studies revealed that for each doubling of the tumor volume, the hypoxic fraction increased by four percentage points.

**Conclusion:**

This study shows correlations between primary tumor volume and hypoxic volume as well as primary tumor volume and the hypoxic fraction in patients with HNSCC. The findings suggest that not only do large tumors contain more cancer cells, they also have a higher proportion of potentially radioresistant hypoxic cells. This knowledge can be important when individualizing RT.

## Highlights

• Primary tumor volume and hypoxic volume has previously not been convincingly related.
• 367 patients with head and neck squamous cell carcinoma from 21 different studies using hypoxia-PET
• The hypoxic volume increased significantly with primary tumor volume.
• In larger tumor the hypoxic fraction was significantly higher than in smaller tumors.

## Introduction

Head and neck malignancies include cancer of the lips, oral cavity, larynx, pharynx, paranasal sinuses, the salivary glands and head and neck cancer of unknown primary. Globally, this group constitutes 4% of all cancers with an incidence of 930 000 cases every year with 80–90% of the tumors being squamous cell carcinomas (HNSCC) [1]. Radiation therapy (RT) is one of the cornerstones in the curative setting, with concurrent chemotherapy for high-risk patients [2].

Hypoxia within tumors is a negative prognostic factor associated with poor clinical outcome [3], [4]. Hypoxia is a result of an imbalance between supply of, and demand for oxygen, and is present in about 60% of all head and neck tumors [5], [6]. Methods to determine hypoxia include invasive Eppendorf pO_2_ histography, through indirect estimations by using RNA sequencing data, and immunohistochemical staining of endogenous (e.g. carbon anhydrase IX, hypoxia-inducible factor-1 α) or exogenous (e.g. pimonidazole) markers [7], [8], [9], [10]. Radiolabeled exogenous markers (tracers) accumulating in hypoxic tissues can be detected by positron emission tomography (hypoxia-PET), providing a non-invasive method to assess intratumor hypoxia [11]. Tracers such as ^18^F-fluoromisonidazole (FMISO) and ^18^F-fluoroazomycin-arabinofuranoside (FAZA) have been shown to be prognostic for patients with HNSCC treated with RT [12]. Hypoxia-PET is often combined with computed tomography (CT) enabling assessment of both the hypoxic volume and the primary tumor volume.

A large primary tumor volume is also a well-known negative prognostic factor for patients with HNSCC [13], [14], [15], [16]. However, the relationship between the hypoxic volume and primary tumor volume has been addressed without any conclusive findings. Using Eppendorf histography, Dunst et al. found a strong correlation between total tumor volume and hypoxic volume, and emphasized a need for newer and more precise methods for measuring hypoxic volume [17]. Chatterjee et al. used hypoxia-PET and showed a correlation between tumor volume and hypoxic tumor volume in a cohort of 18 HNSCC patients [18]. Contrary, Höckel et al. could not show any correlation between tumor oxygenation and tumor size when measuring hypoxia with the Eppendorf histography in uterine cervical cancer [19]. Neither did Stadler et al. find a clear correlation between hypoxic fraction and tumor volume when examining patients with HNSCC using the Eppendorf histograph [3]. Thereby, the relationship between tumor volume and hypoxic volume in human patients has still not been convincingly established.

The aim of our study was therefore to investigate the relationship between primary tumor volume and the hypoxic volume as well as between primary tumor volume and the hypoxic fraction by extracting and compiling published individual patient data from multiple studies using hypoxia-PET in HNSCC patients. The hypothesis was that larger tumors contain both a larger hypoxic volume and a higher hypoxic fraction.

### Material and methods

In this study a scoping review was conducted according to Arksey’s and O’Malley’s methodology from 2005, subsequently clarified and enhanced by Levac, Colquhoun and O’Brien in 2010 [20], [21]. The research question was: “Does larger tumors contain a higher fraction of intratumoral hypoxia?”. The databases PubMed and Embase were searched with the help from a professional librarian using keywords and medical subject headings in combination. Search strategies were related to head and neck cancer, tumor hypoxia and PET-scanning, and a limitation to English language was used (Supplementary Table 1 and 2). The records were imported to the reference management tool Endnote and further on to the screening and data extraction tool Covidence, where duplicates were automatically removed. The remaining titles and abstracts and subsequently full text articles were screened according to the predefined selection criteria (Supplementary Table 3). Several studies gave the appearance of possessing the coveted data for individual patients although not presenting it in the article nor in the supplementary material. In these cases an email to the corresponding address was sent, kindly requesting the individual data. Individual patient data (primary tumor volume/primary gross tumor volume (GTV-T) and hypoxic volume) was extracted from all the relevant articles.

### Statistics

Patient data was imported to Microsoft Excel and the hypoxic fraction (defined as the hypoxic volume divided by the tumor volume) for each patient was calculated (if not already provided). Relationship between primary tumor volume and hypoxic volume was determined by linear regression. To increase the possibility of interpretation of the hypoxic fraction, tumor volumes were transformed to the binary logarithms (log_2_). For each study cohort, hypoxic fraction vs. log_2_ (primary tumor volume) was plotted and the statistical relationship was determined by linear regression. The individual regression slopes and the coefficients of determination (R^2^) were weighted according to cohort size. Comparisons between two groups were determined using the Wilcoxon rank-sum test. In addition, a pooled analysis was conducted. To account for the different thresholds and methods used, we separately normalized the hypoxic fractions in all cohorts by defining hypoxic fraction_Arbitrary Unit_ = 1 at the median tumor volume. This was made by calculating Δy between the fitted linear regression-line and the arbitrary point y = 1 for tumor volume = 25 cm^3^ (median tumor volume). Δy was then added to the individual data points (hypoxic fractions), leaving the regression slope unaffected. A linear regression for the normalized hypoxic fractions was then performed. Different threshold for determining hypoxic fraction was investigated by Spearman’s rank correlation coefficient (ρ) for a subset of patients with available data. Statistical calculations were performed in Microsoft Excel and in R (version 3.6.3) using RStudio (version 1.2.5042). P-values < 0.05 were determined statistically significant.

## Results

### Data acquisition

**Fig. 1:**
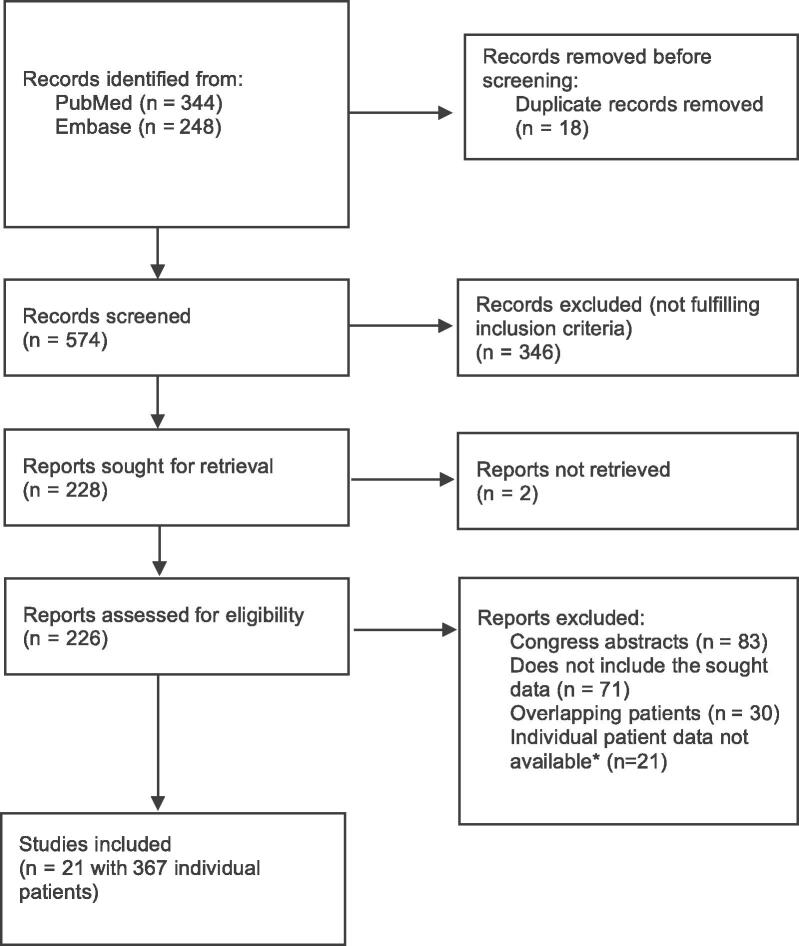
Flow chart of the study selection process. * Individual patient data was requested by e-mail to 25 corresponding authors and resulted in 18 non-responders, 3 responders but data not provided, and 4 authors provided data (of which 1 had overlapping data and not included).

The search in the databases PubMed and Embase resulted in 344 and 248 records respectively which added up to 592 records in total (Fig. 1). Individual data was extracted from 21 articles who met the preset criteria, with a total number of 367 individual patients (Table 1). In three of the patients the hypoxic volume was larger than the primary tumor volume, generating a hypoxic fraction > 1. For these patients the hypoxic fraction was set to 1. In three studies the volumes measured could concern either the primary tumor or lymph nodes, therefore a decision was made to exclude these studies from the pooled analysis. The same was done for a study presenting tumor SUVmax / muscle SUVmean (T/M) instead of hypoxic volume. Finally, 17 studies with a total of 323 individual patients were included in the pooled analysis.

**Table 1:** Characteristics of the 21 studies.

							**Hypoxic volume vs.** **tumor volume**			**Hypoxic fraction vs. log_2_(tumor volume)**				
**Author**	**Year**	**# of patients**	**Tracer used**	**Cut-off**	**Range GTVT (min**–**max)**	**Range hypoxic fraction**	**Linear regression coefficient**	**P-value**	**R^2^**	**Linear regression coefficient**	**P-value**	**R^2^**	**REF**	
Silvoniemi, A	2018	10	EF5	TMR threshold 1.5	8.9–73.5	0.01–0.43	0.403	0.002	0.70	0.0615	0.21	0.19	[43]	
Mönnich, D	2017	21	FMISO	TMR threshold 1.4	15.9–209.3	0–0.71	0.104	0.09	0.10	−0.0327	0.54	0.020	[44]	
Kazmierska, J	2020	35	FMISO	TMR treshold 1.6	0.2–174.3	0–0.14	0.043	<0.001	0.27	0.0075	0.022	0.15	[45]	
Löck,S	2019	42	FMISO	TBR treshold 1.6	5.06–177.85	0–1	0.307	<0.001	0.36	0.0726	0.056	0.09	[46]	
Zegers, C	2016	20	HX4	TMR treshold 1.4	2.4–46.6	0–0.32	0.132	0.008	0.29	0.0377	0.049	0.20	[47]	
Bollineni, V. R	2014	6	FAZA	TBR treshold 1.4	26–50	0.05–0.85	1.320	0.049	0.58	0.6382	0.068	0.61	[48]	
Chang, J	2013	8	FMISO	TMR treshold 1.5	14.5–52.4	0.05–0.16	0.088	0.027	0.52	−0.0142	0.56	0.06	[49]	
Grosu, A	2007	18	FAZA	TMR threshold 1.5	18.8–115	0–0.51	0.290	0.007	0.34	0.0541	0.12	0.14	[50]	
Komar, G	2014	22	EF5	TMR of 1.5	0.98–45	0–0.998	0.089	<0.001	0.72	0.1053	0.026	0.22	[51]	
Lehtiö, K	2004	19	FETNIM	N/A	1.4–401.6	0.095–0.64	0.659	<0.001	0.95	0.037	0.14	0.13	[52]	
Lin, Z	2008	7	FMISO	TBR threshold 1.3	23.45–140.8	0.03–0.48	0.417	0.21	0.15	0.0429	0.52	0.09	[53]	
Saksø, M	2020	40	FAZA	TMR threshold 1.6	1.6–144.6	0–0.84	0.086	<0.001	0.37	0.0151	0.45	0.02	[54]	
Servagi-Vernat, S	2015	12	FAZA	TMR ratio 1.6	2.4–73	0–0.54	0.497	<0.001	0.72	0.0873	0.021	0.43	[55]	
Simoncic, U	2017	6	FMISO	TMR threshold 1.4	11.6–48.5	0.01–0.72	0.418	0.22	0.17	−0.133	0.48	0.13	[56]	
Bittner, M	2016	16	FMISO	TMR threshold 1.4	9–99	0.05–0.99	0.754	<0.001	0.88	0.0068	0.94	0.004	[57]	
Nehmeh, S	2021	18	FMISO	TBR treshold 1.2	3.9–34.9	0.003–1	0.657	<0.001	0.74	0.1164	0.28	0.07	[58]	
Sato, J	2018	23	FMISO	TMR threshold 1.25	0.3–36.1	0–1	0.153	<0.001	0.58	−0.0229	0.37	0.04	[59]	
		**323**					**0.27**§	**<0.001**§	**0.46**§	**0.045**†	<0.001*	**0.12**†		
														**Reason for exclusion**
Dirix, P	2009	12	FMISO	TBR treshold 1.2	14.5–85	0–0.46	0.095	0.17	0.09	−0.0307	0.603	0.028	[60]	lymph nodes or primary tumor volume
Henriques de Figueiredo	2015	10	FMISO	See publication	306–518**	0.01–0.14	0.07	0.31	0.02	0.0048	0.926	0.0011	[61]	lymph nodes or primary tumor volume
Zegers, C	2015	7	HX4	TBR treshold 1.2	2.6–79.9	0–0.15	0.05	0.01	0.70	0.0053	0.739	0.0241	[62]	lymph nodes or primary tumor volume
Minagawa, Y	2011	15	62Cu-ATSM		1.2–76.5	1.61–10.94***				1.9564	0.168	0.1406	[63]	No volume available

Original and analyzed data for the 21 included studies. For each study cohort, the statistical relationship between primary tumor volume and hypoxic volume (light grey columns) as well as between primary tumor volume (binary logarithm) and hypoxic fraction (dark grey columns) were determined. The bottom four studies were excluded from the pooled analysis. GTVT: primary tumor volume, TMR: tumor-to-muscle ratio, TBR: tumor-to-blood ratio.

**Fig. 2:**
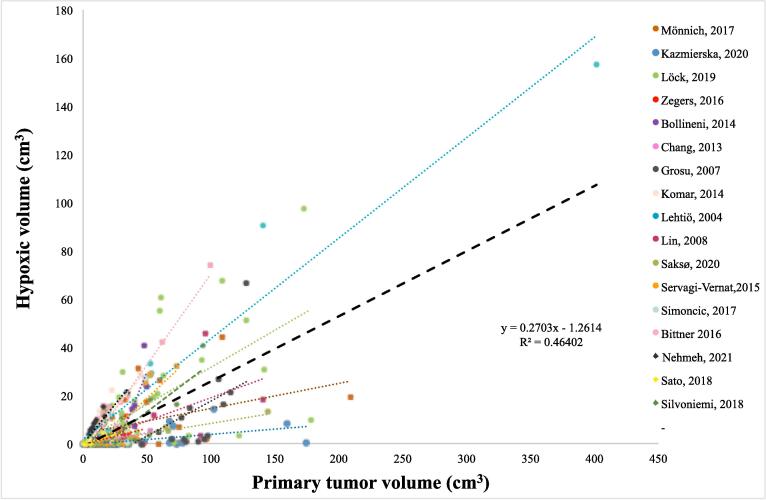

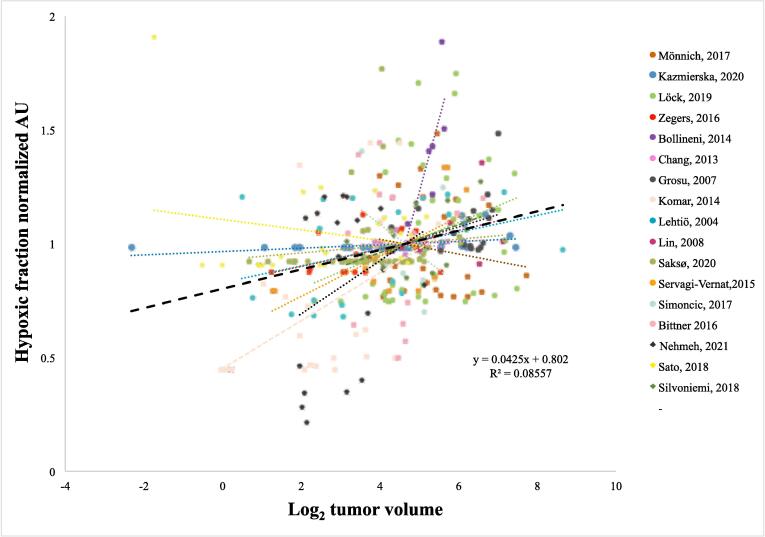
**A)** The hypoxic volume determined by hypoxia-PET as a function of tumor volume for the 17 cohorts included in the pooled analysis. Every cohort is presented in a unique color, and each point represents one individual patient. Lines denote the linear regression of each cohort and thick black dashed line illustrates the linear regression of 323 patients (with regression coefficients in the figure, *P <.001*)*.*

* Result of the pooled analysis of normalized data according to Fig. 2B.

** Values representing clinical target volume (CTV) instead of GTVT.

*** (T/M) tumor SUVmax/muscle SUVmean.

§ Result of the pooled analysis.

† Sum of individual analyses, weighted according to cohort size.

**Table 2:** Correlation between hypoxic fraction and tumor volume for different thresholds to define hypoxia. Values in subscript (_1.4-2.0_) denote the tumor-to-muscle ratio (TMR) for defining hypoxia. Data for 114 patients from [12] including the data from [44], [46], [55] and 39 additional patients.

	Spearman correlation	P-value
Hypoxic fraction_1.4_	0.34	**<0.001**
Hypoxic fraction_1.6_	0.37	**<0.001**
Hypoxic fraction_1.8_	0.38	**<0.001**
Hypoxic fraction_2.0_	0.42	**<0.001**

### Relationship between primary tumor volume and hypoxic volume

Hypoxic tumor volumes as determined by hypoxia-PET was correlated to primary tumor volume (Fig. 2A). Using linear regression, a significant positive correlation between hypoxia-PET determined hypoxic volume and primary tumor volume for the 323 included patients was found (*P <.001, R^2^ = 0.46*). Within the individual studies, significant correlations were found for 15 out of 17 studies (Table 1). Sensitivity analyses by dichotomizing the cohort at different primary tumor volumes and separately analyzing smaller and larger tumors for correlations between primary tumor volume and hypoxic volume revealed similar and significant results throughout (e.g. for primary tumors smaller or larger than 10 cm^3^ the regression slope was 0.29 and 0.28, respectively with *P <.001* in both subgroups).

### Relationship between primary tumor volume and hypoxic fraction

We next investigated the hypoxic fraction in relationship to primary tumor volume. Four of the 17 individual studies showed a positive significant correlation between primary tumor volume and hypoxic fraction (Table 1 and Supplementary Figure S1). In the weighted analysis of all studies a positive correlation between hypoxic fraction and primary tumor volume was found. For each doubling of the primary tumor volume, the hypoxic fraction increased by an average of four percentage points, with a weighted R^2^-value of 0.12. The median hypoxic fraction for patients with a primary tumor volume less than median was 6% (interquartile range [IQR] 0–25) compared with 13% (IQR 2–39) for patients with a primary tumor volume equal to or larger than the median tumor volume (*P <.01*). Moreover, in the pooled analysis of normalized hypoxic fractions, a correlation between hypoxic fraction and primary tumor volume was found with a positive slope of 0.042 (95% confidence interval 0.027–0.058, *P <.001*), corresponding to an average increase of the hypoxic fraction by four percentage points for each primary tumor volume doubling (Fig. 2B). Likewise, the non-normalized pooled analysis showed a positive relationship between hypoxic fraction and primary tumor volume (Supplementary Fig S2). In a subset of 114 patients the correlation between hypoxic fraction and tumor volume was investigated at different thresholds for defining hypoxia (i.e. tumor-to-muscle ratio 1.4–2.0). Significant correlations were found for all hypoxia-defining thresholds, with a tendency towards stronger correlations for higher thresholds (Table 2).

## Discussion

In this pooled analyses of 323 HNSCC patients from 17 different studies using hypoxia-PET we have found a correlation between the primary tumor volume and the hypoxic volume. Moreover, the hypoxic fraction correlated with the primary tumor volume, and increased significantly with increasing primary tumor volume. Thereby, patients who present with a large tumor have, in general, both a larger volume of hypoxic cells as well as a higher proportion of hypoxic cells compared to patients with a smaller tumor.

The present results add substantial knowledge to earlier diverging findings. Similar to the current findings, positive correlations between tumor volume and hypoxic volumes have been found using hypoxia-PET and for Eppendorf histography [17], [18], [22]. In contrary, Saksø M. et al. did not observe any correlation between hypoxia and tumor volume in human head and neck cancer when using both Eppendorf histography and FMISO-PET [23]. Important to note is that their measurements were made in single lymph nodes rather than in primary tumors, which may have affected the result. Neither did Stadler et al. find a clear correlation between tumor volume and hypoxic fraction using Eppendorf histography [3]. These measurements were performed either in the primary tumor or a lymph node metastasis for each patient, which potentially could have affected the outcome. Vaupel et al. did not observe any correlation between the occurrence of hypoxia and tumor size (comparing T1-2 with T3-4 tumors) when using Eppendorf histography in breast cancer tumors, however these observations were only made in a small cohort of 15 patients [24].

A relationship between tumor volume and hypoxia was demonstrated in animal models already in the 60/70′s [25], [26], [27], although the presence of tumor hypoxia and the relationship to tumor volume may be cell line dependent [28], [29]. For six different squamous cell carcinoma models, no relationship between tumor volume and hypoxic fraction was found when hypoxia was assessed after ten fractions of RT [30]. The current study suggests a relationship between tumor volume and tumor hypoxia before start of RT in human HNSCC. Considering that hypoxic tumor cells are more radioresistant, an increased number of hypoxic tumor cells is likely to contribute to the poorer outcome seen in larger tumors. Our results suggest that when a tumor doubles in size, the hypoxic fraction increases by four percentage points.

Several imaging parameters are obtained through hypoxia-PET and consensus for its use is lacking. Cut-off values for defining hypoxic volumes (e.g. 1.4 or 1.6 times the background level in muscle) and normalization methods (e.g. comparing to background level in blood, muscle or cerebellum) differ between studies. Parameters such as SUV_max_ (the maximum of the Standardized-Uptake-Value) and presence of tumor-to-muscle ratio > 2 have proven informative [12], [22]. Image acquisition is typically obtained through static scans, but dynamic scans can provide additional information of perfusion and tracer retention [31]. Hypoxia-PET measured after 1–2 weeks of RT could be more informative compared with before RT [22], [32]. In addition, several tracers with different characteristics are available. Thereby, the optimal usage of hypoxia-PET in HNSCC is yet to be defined. Encouragingly, the prognostic value of hypoxia-PET was recently confirmed in a large meta-analysis, and the two most commonly used tracers (FMISO and FAZA) were found to provide equivalent results [12]. One of the advantages of using hypoxia-PET is its ability to non-invasively visualize hypoxic volumes, enabling direct comparisons with CT-determined tumor volume. Eppendorf histography has been extensively used in the past and can be sampled through tumors, thus providing spatial distribution of hypoxia, but requires an invasive procedure [17], [24]. RNA-sequencing using hypoxic profiles can be used to identify hypoxic tumors and have been shown to be prognostic for patients with HNSCC treated with RT [33], [34]. However, no spatial information is provided. Interestingly, an interaction between CT-determined tumor volume and RNA-seq-based hypoxic profiles was described by Linge et al., and the gene profile was only prognostic for patients with small tumors [33].

There are a number of limitations in the current study. First, being retrospective, this study naturally has a lower degree of evidence. We strived to minimize the risk for selection bias by using beforehand defined inclusion- and exclusion criteria. The cohorts used different tracers and assessment methods leading to heterogeneity in the pooled analysis. Despite this heterogeneity, a statistically highly significant relationship was found in the pooled analysis. Moreover, the study focuses on hypoxia in primary tumors and relies on the volumes as identified in the original target delineations. Hypoxia-PET assessments may underestimate hypoxia in small tumors, which would affect the current analyses [35]. We therefore conducted the sensitivity analyses described above, with similar correlations for small and large tumors. Inherent disabilities, such as partial volume effects, for detecting hypoxia with hypoxia-PET in smaller tumors might still be present and would then contribute to inflate the current results [36]. It has previously been shown that human papillomavirus (HPV)-positive cell lines respond differently to hypoxia [37], and a recent clinical trial investigated the use hypoxia-PET to de-escalate RT for patients with HPV-positive oropharyngeal HNSCC [38]. We could, however, not investigate the subgroup of HPV-associated HNSCC since HPV-status was only available for a small subset of patients. It should be noted that the term hypoxia refers to features identified by the specific hypoxia-PET tracers and are therefore only surrogate markers of the underlying hypoxia The analyses do not investigate any relationships to clinical outcome after RT. Lastly, the results refer to hypoxia before start of RT, and changes during RT or by physiological changes over time are outside the scope of the current study, and it could be noted that hypoxia measured during RT might provide more prognostication than baseline hypoxia [22], [32].

Looking into the future, our result may be useful for individualizing RT in HNSCC-patients. Since hypoxia refers radioresistance, treatment intensification such as dose-escalation or addition of hypoxia-sensitizing agents could be indicated for patients with larger tumors. We have previously described that intensified RT was more beneficial in patients with large tumors [16], [39]. Similar results were found in other HNSCC and lung cancer trials [40], [41], [42]. Our present results could suggest that the underlying mechanism for the increased efficacy of intensified RT in large tumors might be related to tumor hypoxia. In addition, since hypoxia-PET is relatively expensive, time consuming and associated with potential patient discomfort due to long imaging and post acquisition times, primary tumor volume might be used as a screening method for further hypoxia imaging.

## Conclusion

This study shows significant positive correlations between primary tumor volume and both hypoxic volume as well as hypoxic fraction in human head and neck cancers. The findings suggest that not only do large tumors contain more cells, they also have a higher proportion of potentially radioresistant hypoxic cells. This knowledge may be of value in the development of a more individualized radiation therapy. Our findings will need to be validated in another cohort and by using other methods to assess tumor hypoxia.

## Funding

Governmental research funding (Yngre ALF). Mrs Berta Kamprad Foundation (grant no FBKS 2020–19-301).

## Declaration of Competing Interest

The authors declare that they have no known competing financial interests or personal relationships that could have appeared to influence the work reported in this paper.
