# Supplementary material for: Hypoxia in relationship to tumor volume using hypoxia PET-imaging in head & neck cancer – A scoping review
Source: Clin Transl Radiat Oncol. 2022 Jun 15;36:40–6. doi: 10.1016/j.ctro.2022.06.004 (PMC9234341; doi:10.1016/j.ctro.2022.06.004)
Supplement: Supplementary data 1 [file mmc1.docx]

## Supplementary material

**Supplementary figure 1:** Individual regression slopes and coefficients of determination for the different patient cohorts.**** ****

**
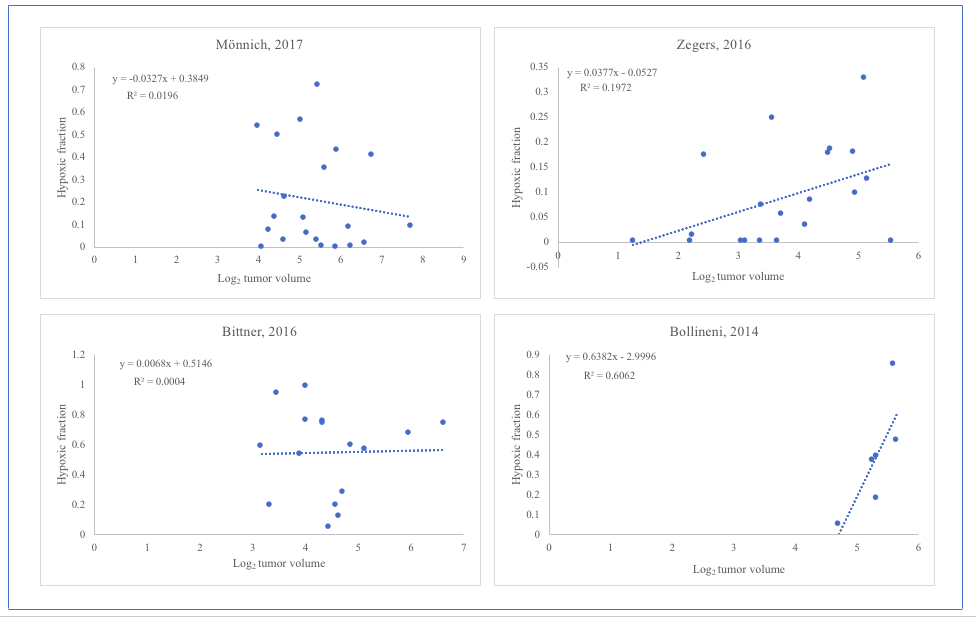
**

**
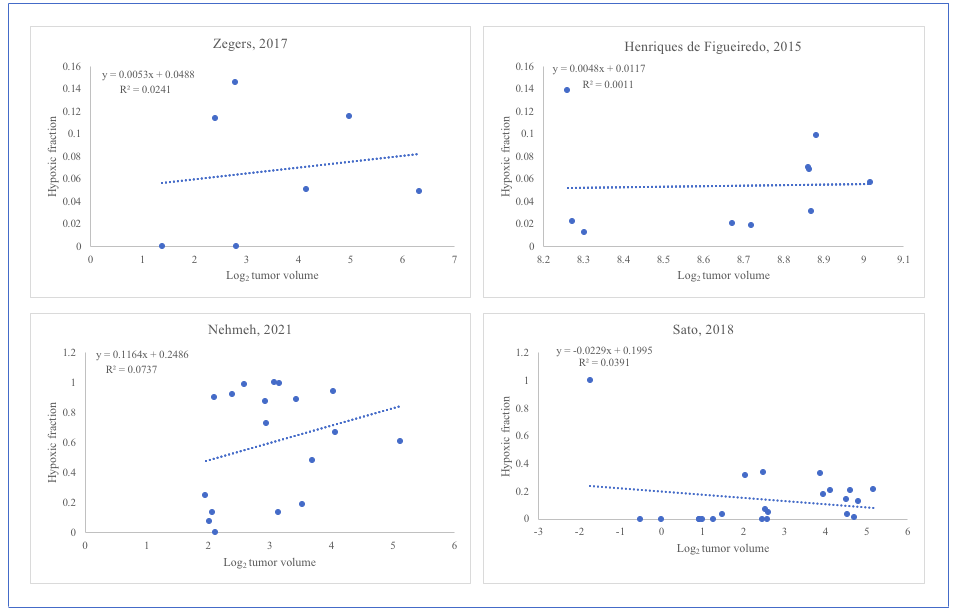
**

**
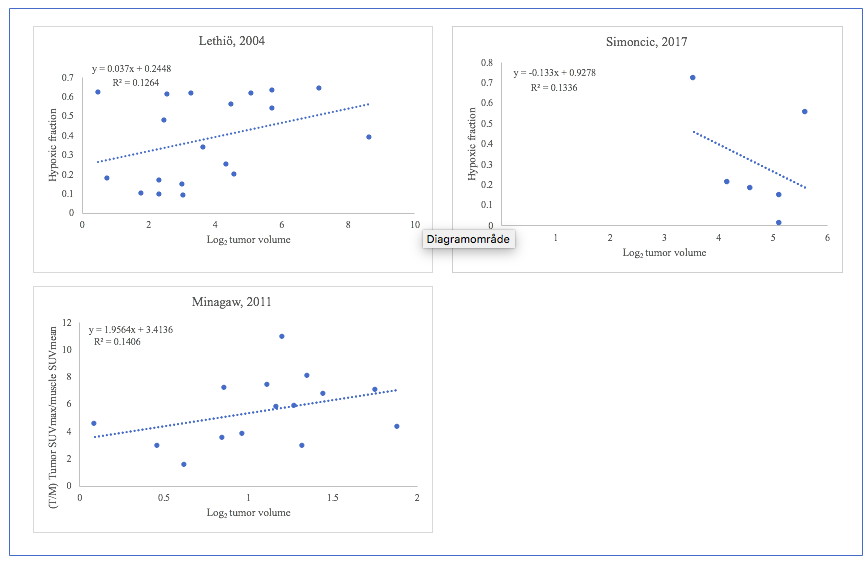
**

**Supplementary figure 2:** Non-normalized hypoxic fraction as a function of tumor volume for the 17 cohorts included in the pooled analysis.

**Supplementary Table 1:** Search strategy in PubMed.

| **PubMed 2021-09-08** | **Number of records** |
| --- | --- |
| **#1** (((tumor hypoxia[MeSH Terms]) OR (tumor hypoxia) OR (tumour hypoxia) OR (FAZA) OR (FMISO) OR (HX4) OR (hypoxia pet) OR (hypoxia tracer) OR (hypoxia[MeSH Terms]) OR (hypoxia imaging[Title/Abstract]) AND (english[Filter])) | 93 312 records |
| **#2** (((Positron emission tomography[MeSH Terms]) OR (Positron emission tomography)) OR (PET) OR (pet scan*) AND (english[Filter]))) | 14 1721 records |
| **#3** ((((((head and neck neoplasms[MeSH Terms]) OR (scc)) OR (hnscc)) OR (head and neck malignancy)) OR (head and neck cancer) OR (head and neck tumor) OR (head and neck tumour) OR (squamous cell carcinoma) OR (squamous cell cancer) OR (squamous cell tumour) OR (squamous cell tumor) OR (oropharyngeal cancer) OR (Nasopharyngeal cancer) OR (Hypopharyngeal cancer) OR (head[Title/Abstract] AND neck neoplasms[Title/Abstract]) ) OR (oropharynx cancer[Title/Abstract]) OR (hypopharynx cancer[Title/Abstract]) OR (nasopharynx cancer[Title/Abstract]) AND (english[Filter])) | 399 179 records |
| **#4** #1 AND #2 AND #3 | 344 records |

**Supplementary Table 2:** Search strategy in Embase.

| **Embase 2021-09-08** | **Number of records** |
| --- | --- |
| **#1** ('hypoxia'/exp OR 'tumor hypoxia'/exp OR hypoxia:ab,ti OR 'tumor hypoxia':ab,ti OR 'tumour hypoxia':ab,ti OR faza:ab,ti OR fmiso:ab,ti OR hx4:ab,ti OR ef5:ab,ti OR 'hypoxia tracer':ab,ti OR 'hypoxia imaging':ab,ti OR 'hypoxia pet':ab,ti) AND [english]/lim | 192 154 records |
| **#2** ('positron emission tomography'/exp OR 'positron emission tomography' OR 'positron emission tomography':ab,ti OR pet:ab,ti OR 'pet scan*':ab,ti) AND [english]/lim | 261 344 records |
| **#3** ('head'/exp AND 'neck tumor'/exp OR (head:ab,ti AND 'neck tumor':ab,ti) OR (head:ab,ti AND 'neck tumour':ab,ti) OR (head:ab,ti AND 'neck malignancy':ab,ti) OR (head:ab,ti AND 'neck cancer':ab,ti) OR (head:ab,ti AND 'neck neoplsm*':ab,ti) OR hnscc:ab,ti OR scc:ab,ti OR 'squamous cell carcinoma':ab,ti OR 'squamous cell cancer':ab,ti OR 'squamous cell tumor':ab,ti OR 'squamous cell tumour':ab,ti OR 'oropharynx cancer':ab,ti OR 'hypopharynx cancer':ab,ti OR 'nasopharynx cancer':ab,ti) AND [english]/lim | 166 290 records |
| **#4** #1 AND #2 AND #3 | 482 records |
| **#5** #4 AND [embase]/lim NOT ([embase]/lim AND [medline]/lim) | 248 records |

**Supplementary Table 3:** Inclusion and exclusion criteria.

| **Inclusion criteria** | **Exclusion criteria** |
| --- | --- |
| - Studies where primary tumor volume and hypoxic volume / or hypoxic fraction could be extracted for the individual patients (alternatively after email-correspondence) - Patients with head and neck cancers - Hypoxic volumes measured by PET-scanning with hypoxia-markers such as FMSIO, FAZA, HX4 (FDG-PET not included) | - Other histology than squamous cell carcinoma - Volumes measured in lymph nodes exclusively |
